# Free Speech at Medical Societies

**Published:** 1897-12-11

**Authors:** 


### FREE SPEECH AT MEDICAL SOCIETIES.

A curious action for libel has recently been decided in the United States Court at New York City, which touches upon an interesting question, in regard to the conduct of the meetings of medical societies. The facts, as given in the New York *Medical News*, are as follows: The plaintiff had an advertisement in the directory, published by the County Medical Society of New York, in the course of which these words were used—"Headache and neuralgia often proceed from latent defects of the eyes, and especially in cases of muscular asthenopia. If present these defects are detected by our method of testing. Proper glasses give permanent relief, and physicians can have a report on any patient he desires." This advertisement gave offence to certain members; and at a regular meeting of the society, Dr. Van Fleet protested against its insertion, which was being sought for the ensuing year. In the course of his remarks, he said that "anybody, not a physician, who undertook to cure headache or neuralgia with glasses or anything else, and accepted remuneration therefore, violated the medical laws of the State, and was a quack; that the society would not allow him to advertise in that way, and it should not allow anyone else to do so." These words were published, and the optician sued the speaker. In answer, Dr. Van Fleet pleaded the truth of his spoken words, and asked for the protection of the Court because of the circumstances under which the remarks were made. The Judge directed a verdict to be entered for the defendant. Commenting on this verdict, the *Medical News* says: "It settles once and for all that a member of the Medical Society of the County of New York has a right to attend the meetings and speak as his sense of duty to the public dictates without fear of suits for libel."

The decision is altogether satisfactory, and it is a matter for much congratulation that New York, now the second city in the world, should possess a medical society so ready to support what is ethically right as to be willing to bear the principal cost of defending a suit of this character, even though it was really occasioned by a criticism of its own proceedings.

